# Evaluating a Web-Based Health Risk Assessment With Tailored Feedback: What Does an Expert Focus Group Yield Compared to a Web-Based End-User Survey?

**DOI:** 10.2196/jmir.2517

**Published:** 2014-01-02

**Authors:** Sandra Vosbergen, Guy R Mahieu, Eva K Laan, Roderik A Kraaijenhagen, Monique WM Jaspers, Niels Peek

**Affiliations:** ^1^Academic Medical CenterDepartment of Medical InformaticsAmsterdamNetherlands; ^2^Academic Medical CenterDepartment of Public HealthAmsterdamNetherlands; ^3^NIPED Research FoundationAmsterdamNetherlands; ^4^Academic Medical CenterCenter for Human Factors Engineering of Health Information TechnologyAmsterdamNetherlands

**Keywords:** health risk assessment, health information systems, qualitative research, evaluation, end users, professional review, designers, optimization

## Abstract

**Background:**

Increasingly, Web-based health applications are developed for the prevention and management of chronic diseases. However, their reach and utilization is often disappointing. Qualitative evaluations post-implementation can be used to inform the optimization process and ultimately enhance their adoption. In current practice, such evaluations are mainly performed with end-user surveys. However, a review approach by experts in a focus group may be easier to administer and might provide similar results.

**Objective:**

The aim of this study was to assess whether industrial design engineers in a focus group would address the same issues as end users in a Web-based survey when evaluating a commercial Web-based health risk assessment (HRA) with tailored feedback.

**Methods:**

Seven Dutch companies used the HRA as part of their corporate health management strategy. Employees using the HRA (N=2289) and 10 independent industrial designers were invited to participate in the study. The HRA consisted of four components: (1) an electronic health questionnaire, (2) biometric measurements, (3) laboratory evaluation, and (4) individually tailored feedback generated by decision support software. After participating in the HRA as end users, both end users and designers evaluated the program. End users completed an evaluation questionnaire that included a free-text field. Designers participated in a focus group discussion. Constructs from user satisfaction and technology acceptance theories were used to categorize and compare the remarks from both evaluations.

**Results:**

We assessed and qualitatively analyzed 294 remarks of 189 end users and 337 remarks of 6 industrial designers, pertaining to 295 issues in total. Of those, 137 issues were addressed in the end-user survey and 148 issues in the designer focus group. Only 7.3% (10/137) of the issues addressed in the survey were also addressed in the focus group. End users made more remarks about the usefulness of the HRA and prior expectations that were not met. Designers made more remarks about how the information was presented to end users, quality of the feedback provided by the HRA, recommendations on the marketing and on how to create more unity in the design of the HRA, and on how to improve the HRA based on these issues.

**Conclusions:**

End-user surveys should not be substituted for expert focus groups. Issues identified by end users in the survey and designers in the focus group differed considerably, and the focus group produced a lot of new issues. The issues addressed in the focus group often focused on different aspects of user satisfaction and technology acceptance than those addressed by the survey participants; when they did focus on the same aspects, then the nature of issues differed considerably in content.

## Introduction

Over the last decade, there has been increasing interest in Web-based health applications for the prevention and management of chronic diseases. However, significant problems have been reported with the reach and utilization of these health applications [1,2]. According to Wixom and Todd [3], certain factors influence the use of information technology. For example, users of information technology must find it relatively advantageous, easy to use, and compatible with their beliefs and attitudes [3]. To increase the adoption of an innovation, we need insight into end users’ real-life experiences and the potential issues to be solved. Various formative evaluation methods to collect this kind of information are available.

A previous evaluation of a Web-based health risk assessment (HRA) with tailored feedback, which will be the focus of this study, showed that only 33.7% of the 6790 employees invited to participate in the HRA did so [4,5]. To optimize this HRA, two evaluations were performed independently. After participation in the HRA as end users, user feedback was collected with a Web-based survey, and expert feedback was collected in a focus group with industrial design engineers. These evaluation techniques are often deployed in practice after a system’s implementation, as they are relatively efficient and easy to administer. However, until now little research has been done to support the claim that end-user surveys are the best option to get insight into experiences and issues influencing adoption of health innovations. Our main question was whether an expert focus group would bring up similar issues to those addressed in the end-user survey. The results of the Web-based survey were published previously in [5]. If a focus group with industrial design engineers would come up with similar issues as revealed by the Web-based survey, a strong point would be made for collecting feedback through this method in a pilot implementation. If these issues were solved in a system’s redesign before its full implementation, end users may perceive the innovation as more valuable, ultimately increasing its adoption rate.

Studies in the field of usability have already shown that, along with end users, usability experts can contribute significantly to the development process of applications because they can give a detailed analysis of potential problems [6] and identify their causes [7]. Research in the fields of usability and text evaluation has generally shown that the types of problems revealed in end users’ and experts’ evaluations [7-11] and by different evaluation strategies [8,12-16] only slightly overlap. However, these studies mostly focused on revealing usability or text understanding issues before implementation of a product, in a laboratory setting, or a study setting. Usability is only one aspect that influences the acceptance of a system, as it mainly focuses on system function [17]; whereas, it is the total user experience that influences the final adoption of an innovation through users’ perception of the value and service quality [18]. To enhance the adoption of innovations, we need broad-based information about user experiences that influence their satisfaction with and acceptance of its design post-implementation.

In this study, we compared two commonly used methods to inform the optimization process of a system after its implementation: end-user surveys and expert focus groups. We assessed whether those two methods would produce similar feedback on issues affecting user satisfaction and technology acceptance. Surveys among large groups of end users are generally performed to provide information on end users’ overall satisfaction with a health care innovation and its effect on their health-related behavior [19-21]. These surveys generally include an open-text field to provide additional space for users to describe their experiences. Industrial design engineers, instead of Web design evaluators, were invited as focus group members because their expertise lies in applying approaches focused on improving user experiences and exploring—and finding solutions to—problems in a broad spectrum of design aspects ranging from esthetics and ergonomics to human-product interaction, user needs research, and usability [22]. Therefore, they could provide a broad view of issues regarding the complete HRA user experience, including those parts not directly associated with the Web-based part of the HRA. Focus groups were chosen because industrial design engineers are familiar with this technique as most of their work is performed in teams. Also, focus groups are a well-established technique in market research for the design and optimization of new innovations (eg, [23]), as well as for human factor research and usability evaluation [24].

As a common denominator to lump the results of the evaluations of industrial design engineers and end users together, we used constructs from user satisfaction and technology acceptance theories [3]. These theories describe various domains that potentially influence the end user’s attitude towards a system such as information quality, service quality, organizational quality, system quality, end users’ outcome expectations, ease of use, and usefulness of a product.

The aim of this study was to assess whether industrial design engineers in a focus group would address the same issues as end users in a Web-based survey when evaluating a commercial Web-based HRA with tailored feedback. Additionally, we aimed to gain insight into the kinds of issues addressed in the focus group and end-user survey, using constructs of user satisfaction and technology acceptance. This study is a comparison of the yield of two evaluation techniques used as they commonly are in practice, not as a traditional experiment.

## Methods

### Description of the Web-Based Health Risk Assessment

The focus of this study is on the evaluation of a Web-based HRA program. HRAs collect health-related data that are used to evaluate an individual’s health status and health risks, typically screening for risk factors of chronic diseases and identifying related health behaviors [25]. They can be used to assess average health risks in a group or population or to provide tailored feedback on health risks to individuals in an effort to help them reduce their risks. In the latter case, the primary goal is to prevent high-incidence chronic diseases such as coronary heart disease, cancer, and diabetes. HRAs used in the workplace have shown promising results in the prevention of chronic diseases [25-28]. Nowadays, HRAs are often disseminated via Web-based technology. Web-based HRAs can reach more people, thus facilitating the impact of HRAs on public health [29].

The Web-based HRA with tailored feedback evaluated in this study consisted of four components completed in the following sequence: (1) a Web-based electronic health questionnaire, (2) biometric evaluation, (3) laboratory evaluation, and (4) an individually tailored feedback report based on the results of the first three components provided in a Web-based environment. The aim of the health questionnaire was to capture data on a participant’s health and lifestyle. It consisted of questions concerning sociodemographic variables, family and personal medical history, health complaints, psychological functioning, lifestyle behavior, and perceived health perception. The biometric data that were collected consisted of a participant’s weight, height, waist circumference, and blood pressure measurements. Blood and urine samples were collected by certified health professionals at predetermined sites and analyzed at a lab. All procedures and components of the HRA were exactly the same for both end users and industrial design engineers, except for the way in which participants’ data on weight, height, waist circumference, and blood pressure was collected. The end users’ biometric data was collected at the predetermined sites by certified health professionals at the same time as the blood and urine samples. Industrial design engineers collected this data themselves at home using a toolbox provided by the HRA.

The tailored feedback report was automatically generated by a computerized decision support system (CDSS) after the participant had completed all HRA components in full. The report described the individual’s overall health risk (expressed by a compass metaphor and a four-color system), results on health risks in five categories (behavioral, psychological, physical, personal medical history/family risk, and work-related) distinguished by a color system per category, an explanation of the risks and potential benefits of taking preventive action, and actionable advice for improving a participant’s health. When the participant’s health was “seriously off-track” (the color red), a referral to a health professional was provided for further medical evaluation and treatment. A more extensive description of the HRA, including examples and screenshots, can be found elsewhere [5].

### Design and Study Population

**Figure 1:**
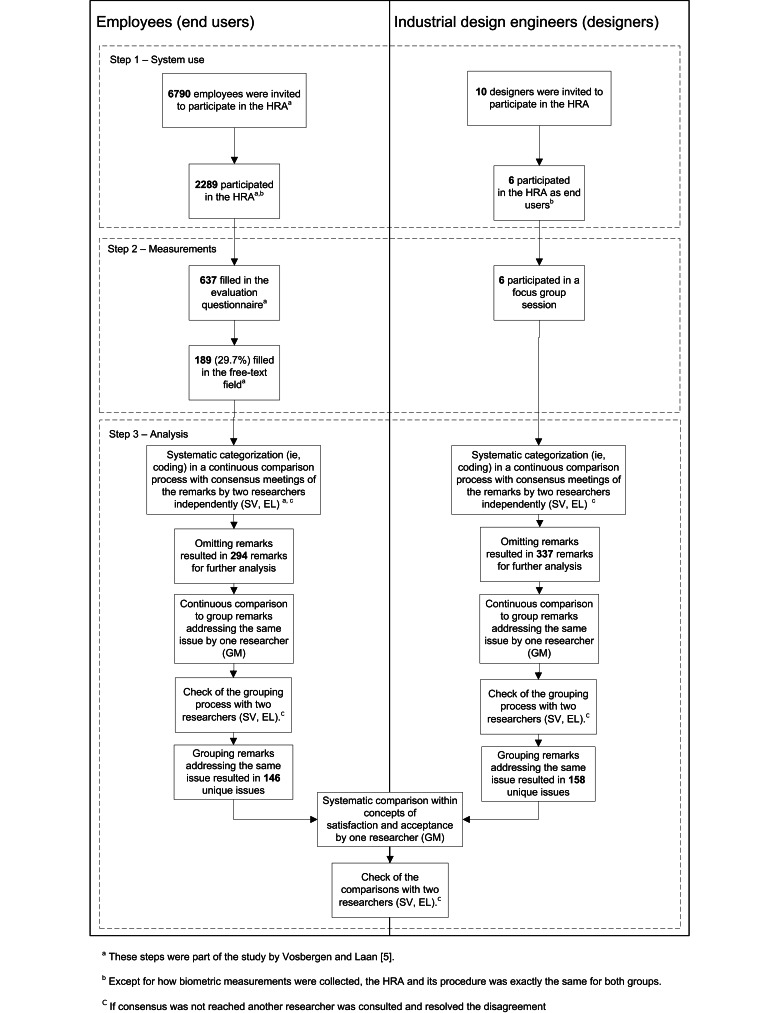
Study design and participant flow.

The study design is depicted in Figure 1. For the end user evaluation, the HRA was implemented in seven Dutch companies as part of their corporate health management strategy between 2007 and 2008. Employers initiated the HRA by sending invitations to their employees. A single reminder was sent 2 weeks later. These invitations consisted of a description of the HRA and a notification that participation was voluntary, paid for by the employer, all personal data would be treated confidentially, and no results would be shared with their employer or any other party. A Web-based survey was subsequently used to evaluate their experiences with the HRA. Participating employees are referred to as “end users”.

Ten industrial design engineers were recruited via the researchers’ own network to acquire the recommended group size of 6-10 people in focus group research [30]. All invited industrial design engineers had obtained a bachelor’s and master’s degree from the Faculty of Industrial Design Engineering at the Delft University of Technology (DUT) or were finishing such a master’s degree. The bachelor and master programs at the Faculty of Industrial Design Engineering are a mixture of theory concerning various aspects of design and applied design projects in collaboration with the business community. The designers were invited to participate in the same HRA for evaluative purposes in 2009. The invitation included a description of the HRA, the goal of the research, and explained that they were first expected to take part in the HRA as an end user and then jointly evaluate the HRA in a focus group session afterwards. It also explained privacy aspects and that participation was free of charge. We refer to the industrial design engineers as “designers”.

Before conducting the actual evaluation, all survey participants and focus group members participated in the Web-based HRA with tailored feedback as end users. To complete the HRA, only one use scenario is possible. It is therefore unlikely that the two evaluator groups differed in the time spent completing the HRA. No further instructions were given on what aspects of the program to evaluate or focus on during the evaluation because we wanted to evaluate the HRA in the broadest sense and aimed to see what issues both evaluation techniques would yield naturally. End users and designers did not have any experience with this type of program, as the HRA program evaluated in this study is the first in its kind in the Netherlands.

### Measurements

#### End Users

Six weeks after having participated in the HRA, end users received an evaluation questionnaire by email. This two-part questionnaire consisted of a quantitative and a qualitative section. The first part assessed end users’ satisfaction with the HRA and the initiation of health behavior change after participation. In this section, respondents were asked to appraise the different components of the HRA and the overall satisfaction with the HRA on an ordinal ranking scale. Results concerning end users’ satisfaction with the HRA are reported elsewhere [5]. Participants were also asked whether they initiated health behavior changes after participation and which behaviors they changed. These results are reported in [4].

The current study focuses on the free-text comments in the second part of the questionnaire. This free-text field was preceded by the following text: “It’s possible that some things were not contained in the above questions, or that you weren’t able to express these things as you would have liked. If this is the case, please enter them below.” The outcomes of the detailed analysis of the remarks that were made by end users in this free-text field can be found in [5].

#### Designers

After completing the HRA as an end user, designers were asked to participate in a focus group discussion. The focus group meeting was led by a moderator and introduced as follows: “I actually want to start with your general impression of the HRA itself, for example how the process works, its strong and weak points. If you already have improvements in mind, you can also mention them.” The entire focus group session was videotaped for evaluative purposes after receiving the participants’ consent and was subsequently transcribed verbatim.

### Data Analysis

Qualitative data analysis was aimed at comparing issues addressed in the end-user survey and in the designer focus group. To achieve sufficient reliability, our main goal was to code the remarks made in both evaluations exactly the same. To do so, an iterative coding, grouping and comparison process was applied.

A codebook was constructed to analyze all textual remarks made by end users and designers. This codebook was based on the domains and topics described in Table 1 of the article by Wixom and Todd (p. 88 [3]), which integrates constructs from user satisfaction and technology acceptance theories. These theories were developed to understand and evaluate factors explaining users’ perceptions about information systems to assess actual usage of these systems. We decided to use all topics and domains, rather than the framework of Wixom and Todd [3], to evaluate the HRA in the broadest sense. The codebook was used to analyze all remarks made by end users and designers alike. The final codebook can be found in Multimedia Appendix 1.

The end users’ remarks had already been categorized using the codebook for the study by Vosbergen and Laan et al [5]. In this section, we describe only the methods we applied for qualitative analysis of the transcripts of the focus group session with the designers, since remarks of both the end user and designer group were analyzed in exactly the same way. At the start of the analysis, the codebook contained all domains and topics described in Table 1 of Wixom and Todd [3]. During analysis, interpretations of these domains and topics were specified to the HRA. Two researchers (SV and EL) independently categorized remarks according to the following topic schemes: (1) the component of the HRA addressed, (2) the domain and topic from the codebook, and (3) whether the remark was positive, negative, neutral, or a recommendation. Based on these topic schemes, the remarks in the transcripts were divided into individual remarks during coding. For example, if a designer’s remark was about both the electronic health questionnaire and the feedback report, it was divided into two separate remarks. If the remaining part of the transcript covered another topic from the codebook, the remarks in the transcript were divided again based on these topics.

End user and designer remarks were categorized in an iterative process, in which independent coding by the two researchers was alternated with consensus meetings. These meetings were aimed at resolving discrepancies so that remarks of end users and designers would be coded exactly the same. During these meetings, consensus was sought by comparing and discussing the individual codes assigned to every text excerpt until all discrepancies were solved and the categorizations and interpretation of the codebook were exactly the same. If consensus could not be reached, a third researcher (NP for end users, GM for designers) resolved the disagreement. After every change in interpretation of the codebook or in categorization of the remarks, all previously categorized data was checked for consistency with the decisions made during the preceding meeting. There were no remarks that did not pertain to any of the domains of the original codebook. If a remark did not pertain to one of the topics within the original codebook, a topic from another domain was considered or an appropriate new topic was added to the codebook. Adding a (new) topic to the codebook was always done in mutual agreement during a consensus meeting. The topics that were copied or added can be found in Multimedia Appendix 1.

Different remarks may or may not address the same issue. To identify the set of unique issues that were addressed, remarks that were syntactically different but semantically similar, meaning that they addressed the same issue, were grouped together. This was done in a bottom-up fashion. Initially, all remarks were viewed as singleton groups (ie, containing a single remark). Subsequently, groups of remarks addressing the same issue were joined. This was done recursively until all groups addressed different issues. For example, all remarks about printing the HRA’s feedback report (eg, “Too bad the results can’t be printed out so you can read through them at a time and place of your choosing”, “Can’t print out recommendations for healthier eating, a real pity...”) were placed in one group. Remarks that had been categorized with different domains or topics were never merged into a single group, unless the coding was found to be inconsistent during the grouping process (see below). Remarks that had been categorized with the same domain and topic could be merged into one group, but only if they addressed the same issue. The grouping was performed separately for remarks made by end users and remarks made by designers.

Remarks about the procedures for collecting biometric data measurements were omitted from the dataset prior to analysis because they could not be compared due to differences between end users and designers. The grouping of equivalent remarks was performed by one researcher (GM). As remarks that had been categorized with different domains or topics were assumed never to be equivalent, combinations were first sought within topics. As a reliability check on the categorization of the data, combinations were then sought within domains, and finally across domains (ie, within the topics from user satisfaction and technology acceptance theories). If an inconsistency was found, then discrepancies were resolved in a consensus meeting with three researchers (GM, EL, SV). After this process was completed, 2 researchers (EL and SV) independently assessed the resulting groups of remarks, followed by a consensus meeting to resolve discrepancies. For each of the final groups, one remark was chosen that best portrayed the issue addressed by the group. This representative remark is given to illustrate the qualitative results. Finally, the issues identified in the end-user survey and designer focus group were compared qualitatively on equivalency in the same manner as described previously for the grouping process.

## Results

### Study Population

Figure 1 depicts the flow of the participants through the study and the number of resulting remarks and issues for both groups. End user response to the evaluation questionnaire was 27.8% (637/2289 participants in the HRA); 29.6% (189/637) of those end users made one or more remarks in the free-text field. Participants were mainly male (386/637, 60.6%) and an average age of 46.49 years (SD 8.76); 36.1% of these participants were aged 50 or older. 48.2% (307/637) of participants had a high education level, 30.0% (191/637) an intermediate education level, and 21.8% (139/637) had a low education level. Except for age, there were no significant differences in sociodemographics between respondents who made remarks in the free-text field compared to those who did not. Respondents who made remarks were nearly 2 years older (mean 47.8, SD 8.1 years) than those who did not make remarks (mean 45.9, SD 9.0 years). More information on end users’ backgrounds and responses to the evaluation questionnaire is available in Vosbergen and Laan et al [5].

Furthermore, a total of 6/10 recruited designers participated in the focus group. Of those, 5 obtained their master’s degree in the 12 months before the start of the study and the other was in the final phase of obtaining a master’s degree. One designer obtained a degree in Strategic Product Design (corporate strategy and opportunities to develop sound product development portfolios), 4 obtained a degree in Design for Interaction (the way in which people interact with products; designing products appropriate to users’ needs and expectations), and one was finishing the master curriculum Integrated Product Design (systematic approach of product design; designing innovative products and service combinations). Four of the designers had gained experience with either product design, product evaluation research, or sales and project management in a company during their studies.

Four of the six designers completed all steps of the HRA within 1 month. The other 2 were unable to complete the HRA before the focus group session due to time limitations (n=1) and fear of the needles used to collect the blood sample (n=1). Both designers, however, did complete all steps in the HRA; however, they did not receive their own risk profile and recommendations but received a fictitious health risk profile and fictitious health recommendations instead to be able to complete the evaluation. The designers’ focus group session lasted around 3.5 hours.

### Issues Identified in the End-User Survey and Designer Focus Group

#### Methods

After omitting the remarks about the approach used to collect biometric data, categorization of the free-text field and transcripts resulted in 294 remarks made by end users and 337 remarks made by designers. Grouping together similar remarks revealed 137 unique issues addressed by end users only, 148 unique issues addressed by designers only, and 10 unique issues addressed by both. Representative remarks of the issues that were addressed by both evaluators are found in Multimedia Appendix 2.

Table 1 lists the results of the systematic comparison of the issues identified in the end-user survey and in the designer focus group. In both evaluations all domains were relevant, but some topics were addressed in only one of the evaluations or were not relevant at all (see Multimedia Appendix 1). There were also topics that were addressed in both evaluations, but comparison of the associated issues showed that the nature of these issues were often dissimilar. Below, we first provide a high-level overview of the similarities and differences in issues identified in the evaluations for each of the user satisfaction and technology acceptance domains. Then we provide a more detailed description of these similarities and differences, using representative remarks to illustrate the findings.

**Table 1:** Numbers of unique issues addressed in the end-user survey and in the designer focus group, categorized using constructs of user satisfaction and technology acceptance.

Domain and topic		Total number of issues addressed by end users or designers, n	Issues addressed by end users, n	Issues addressed by designers, n	Issues identified by both groups, n	Percentage of issues in this domain made by both groups, % (n)
**Ease of use**		18	10	10	2	11.1 (2/18)
	Easy to use	11	9	4	2	
	User-friendly	7	1	6	0	
**Information quality**		77	27	54	4	5.2 (4/77)
	Accuracy	7	7	1	1	
	Completeness	18	7	12	1	
	Format	39	9	32	2	
	Language	3	2	1	0	
	Precision	7	1	6	0	
	Volume	3	1	2	0	
**Organizational factors**		54	14	41	1	1.8 (1/54)
	Communication	28	2	26	0	
	Organizational competition	5	5	0	0	
	Documentation	2	0	2	0	
	Error recovery	5	4	1	0	
	Management	8	0	8	0	
	Data security	5	2	4	1	
	Time	1	1	0	0	
**Outcome expectations**		30	26	5	1	3.3 (1/30)
	Accuracy	3	3	0	0	
	Confidence in the system	8	8	1	1	
	Feeling of control	3	3	0	0	
	Expectations	13	11	2	0	
	Health effects	3	1	2	0	
**Service quality**		54	34	22	2	3.7 (2/54)
	Attitude	1	0	1	0	
	Communication with program staff	5	4	1	0	
	Means of input for the HRA	23	10	14	1	
	Processing of change requests	4	4	0	0	
	Relationship with program staff	2	2	1	1	
	Response time	1	0	1	0	
	Schedule of products or services	11	8	3	0	
	Staff support	1	1	0	0	
	Technical competence of program staff	6	5	1	0	
**System quality**		27	10	17	0	0.0 (0/27)
	Accessibility	3	2	1	0	
	Efficiency	13	2	11	0	
	Errors	2	1	1	0	
	Flexibility	3	2	1	0	
	Language	3	0	3	0	
	Tailoring	2	2	0	0	
	Timeliness	1	1	0	0	
**Usefulness**		35	26	9	0	0.0 (0/35)
	Relevancy	8	5	3	0	
	Usefulness	27	21	6	0	
Percentage of issues in all domains made by both groups						3.4 (10/295)

#### Overview of Similarities and Differences per Domain

Issues identified by designers concerning “ease of use” mostly focused on the user friendliness of the system and possible improvements, while those of end users were merely focused on practical issues that were encountered (eg, not being able to print the feedback). Twice as many issues were addressed by designers compared to end users concerning “quality of information”. The majority of these issues addressed by designers focused on information completeness and on the format of information throughout the HRA. End user issues regarding these topics focused on accuracy of the information provided by the HRA. Within the “organizational factors” domain, most of the issues brought up by designers concerned availability of accurate information about the HRA before actually using it. They discussed various aspects of how to motivate potential participants to use the program (eg, Table 2, remark d5). Conversely, within the “outcome expectations” domain, five times more issues were addressed by end users. These remarks mainly concerned the fit of the system to their prior expectations and perceived reliability of the HRA’s feedback. In the “service quality” domain, both groups identified a similar amount of issues (34 for end users, 22 for designers); however, only two issues were addressed by both groups. A majority of the issues identified by designers categorized under this domain focused on methods and data collection tools used to deliver the different components of the HRA and how these related to usefulness of the system (eg, Table 2, remark d6). End users also remarked on the methods and data collection tools used, but only one of those issues overlapped with those of designers. Furthermore, the issues identified by end users covered more topics within this domain. Within the “system quality” domain, a few issues were addressed by both groups for every topic. The eleven designer issues that concerned various aspects of the system’s efficiency domain, however, stand out within this domain. Finally, end users brought up at least 2.5 times more issues in the “usefulness” domain. They focused mainly on the extent to which the HRA actually helped them solve their health problems (eg, Table 2, remark e8).

**Table 2:** Examples of end-users’ remarks and designers’ remarks to illustrate the differences in yield of the two evaluation methods.

End-user remark	Designer remark
Remark e1 After receiving the results, I didn’t really understand the feedback. I got the advice to eat healthier. I actually already started eating healthier food some time ago (and I have indicated this in the questionnaire). Still I received this advice, but I wouldn’t know what else to do. (Information quality/Completeness)	Remark d1 Just explain that there is one way to complete things. People should just…you just have to guide them, because that’s the most useful. It’s also the most efficient. (Information quality/Completeness)
Remark e2 My general practitioner was really unsatisfied with how the HRA works, there is no explanation given about what has been tested precisely et cetera. Due to these results, there have been, according to my general practitioner, needless blood tests via STAR. (Information quality/Accuracy)	Remark d2 What you can also do, you have those five or six subjects in the feedback report, you can also have a compass per health subject. Because then you’ll see a compass at the left top, this compass does this (mimics a compass pointer): for this subject you go wrong and for this one you go well. (Information quality/Format)
Remark e3 The feedback report of the HRA got across “fiercer” on me than it was in fact. It is a good realization and certainly a good provocation to take action. Nevertheless, I had preferred a few things to be expressed more subtle. (Information quality/Format)	Remark d3 Make it a little bit more personal, instead of relating it to some kind of standard. Because now you get: this is healthy, this is you, you fail in this, or you fail on that. But just say something like: This is personal, this is what you are now, and this is what you could do, this, that, et cetera. Instead of, relating it to the mass, you are wrong here and there and there. (Information quality/Format)
Remark e4 The feedback report is clear, but I am wondering whether one can conclude from these limited tests how healthy I live and what my physical condition is. (Outcome expectations/Confidence)	Remark d4 If I were to do it, I would just like my house style to be consistent. (Organizational factors/Communication)
Remark e5 I realize you want the phrasing of the questions to be as clear as possible. In a number of cases, the answers are oversimplified. The actual situation is sometimes far removed from the possible answers and consequently the results also give a different (more negative) picture. (Outcome expectations/Feeling of control)	Remark d5 You have to get someone excited, so you can say that it is a gift to the people. […] And that you, for example, mention somewhere: this package costs X euro, but the government and the employer believe it is important that…Just a story that they know that it is no garbage, but that it is actually really valuable and that they get it for free. (Organizational factors/Communication)
Remark e6 The examination does not have any relation to my work activities. Work-related problems/complaints are insufficiently covered because of this. (Outcome expectations/expectations).	Remark d6 That’s when I thought, I will first fill in the questionnaire before I set to work with measuring my blood pressure. Or was it that I first waited for the lab box, I don’t know anymore. I first want to have everything, you know, to get an overview of what I have to do. (Service quality/Means of input for the HRA)
Remark e7 During a face-to-face talk you could have given a lot more information and clarified things, and also have had a more thorough physical examination. (Service quality/Means of input for the HRA)	Remark d7 With the card you can activate your account and you also have to use the card to perform your measurements (has the card in his hands). Everything you do is stored on your card and if you log in with the card, your data will automatically be stored on the website, on your account. And the parameters, the outcomes of the blood tests are eventually also stored on your card and with this card you can go to the general practitioner and he explains to you what to do. (Service quality/Means of input for the HRA)
Remark e8 I don’t think everybody needs to have an HRA. It upsets people more than anything else and doesn’t give any guarantees at all. It’s useful for (hereditary) diseases in the family. The question remains as to whether this should be done through the employer. (Usefulness/Relevance)	Remark d8 You can immediately register at the Internet. You immediately receive a login, so you can immediately fill in the questionnaire and only if you have completed the questionnaire you receive the home measurement tools. (System quality/Efficient)
Remark e9 Loss of time, drawing conclusions based on length, weight and a few simple Internet-based questions. (Usefulness/Usefulness)	Remark d9 It is ideal to just, as an employee, so to speak not with your Christmas box, but…So you get it from your employer, you think: ‘Hey that employer has exerted himself, it is a subsidized thing.’ I really think it is a good cause. (Usefulness/Relevance)
Remark e10 A polyp has been removed from my intestines on two different occasions. According to the specialist, one of these would certainly have become malignant. (Usefulness/Usefulness)	

#### Similarities

Issues that were found to be similar for both evaluator groups mainly focused on the feedback report provided by the HRA and on various aspects of using the Web-based part of the system. Both groups had some comments on the presentation and communication of the feedback report. They remarked that risk categories and recommendations mentioned in the feedback report could be clearer if a more extensive explanation on how to interpret the results would have been given: “What is required is that you get a printout of all the recommendations along with the referrals to a website or individual. Along the lines of, what is good, what isn’t, and what does it actually mean?” [Designer #4, Information quality/Completeness]

Some also indicated that the feedback was not communicated in a way that optimally motivated them to change their lifestyle behavior: “Because all communication is written (in other words, in the system), it’s easy to disregard any potential recommendations” [End user, female, age 56, Information quality/Format] and “No, but the results also didn’t really provide the motivation to go into them at length” [Designer #6, Information quality/Format].

Some respondents of both groups also felt that the feedback was not inferred from both the answers to the questions and the biometric data, but merely represented their answers to the questionnaire: “Results are based on the answers that are filled in, and not on blood and urine tests” [End user, female, age 31, Outcome expectations/Confidence in the system]. Some end users added that if the HRA was more transparent about the origin of the results (eg, the data and evidence that led to these particular results), this could mitigate these problems. This suggestion was not made by designers.

#### Differences

Table 2 provides some examples of remarks to illustrate differences in yield of the two evaluation methods; a more comprehensive overview can be found in Multimedia Appendix 3. Within the “information quality” domain, designers mainly discussed how health risks and recommendations were displayed. The designers had extensive discussions on various design choices, like the choice of the compass metaphor for displaying the overall health risk of an individual. They found that if a metaphor is used, it should be used throughout the entire system and not just for displaying a single aspect (eg, remark d2). End users also commented on the use of the metaphor, but because they found the feedback displayed through use of the compass too alarming (eg, remark e3). Furthermore, designers also had discussions on ways to clarify the feedback report and present it in a way that might better motivate users to change their health behavior (eg, remark d3).

Both end users and designers addressed issues concerning the information completeness. End users remarked that they felt that the clarification of the values of the blood tests performed, of what to do with the feedback received (eg, remark e1), and the clarification of the tests performed to the general practitioner was unsatisfactory (eg, remark e2). In contrast to end users, designers’ discussions mainly focused on the lack of guidance while completing the various HRA components (eg, health questionnaire, biometric measurements). They felt it was not clear enough in which order these components should be completed. They proposed explaining the single path for completing the entire program in a clear-cut way (remark d1).

The sequence of HRA components was further discussed in terms of efficiency (System quality/Efficiency). Designers indicated that because it was not possible to continue with another HRA component without completing the previous component and because the order of components was not clear, the process was not efficient and would therefore be a barrier for participants to complete the HRA (eg, remark d8). The designers also noted problems with the presentation of the health risks and feedback and discussed potential improvements extensively (eg, remark d3). The end users also identified issues concerning the presentation but added that the feedback report could have contained more practical advice (eg, remark e3).

Within the “organizational factors” domain, designers focused on the “look and feel” of the entire HRA from a marketing perspective, including both the Web-based aspects as well as pamphlets and other materials provided with the HRA. They were both impressed by some aspects of the HRA and disappointed by others. For example, they were impressed by the use of colors but disappointed by the use of stock photos within the Web-based environment. They also discussed the style of the various HRA components and recommended the use of a more consistent house style throughout the components (eg, remark d4).

End users, on the other hand, identified many more issues in the “outcome expectations” domain, which focused particularly on the ways in which the HRA met their expectations (eg, remark e6) and the confidence they had in the system (eg, remark e4). They felt they were unable to enter all the information they deemed necessary to generate the HRA feedback. Some mentioned that the questionnaire was not sufficiently tailored to their personal situations. End users were concerned that this affected the reliability of the provided health risk profile and feedback (eg, remark e5). Consequently, some end users had the desire to consult a health professional and have the opportunity to provide the aforementioned details during a conversation.

Remarks about the need for a clinical follow-up appointment also appeared in the “service quality” domain. In this domain, both end users and designers identified issues about the methods and data collection tools used in the HRA. Some end users wanted a follow-up appointment to discuss the results with a clinician and expected there would be more physical tests in the HRA (Service quality/Means of input for the HRA). In general, these end users preferred a more personal approach and the opportunity to clarify particular aspects of their health (eg, remark e7).

In contrast, designers focused mainly on exploring the opportunities for the future of HRAs with new means of performing the process of assessing one’s health risks and giving feedback. New input methods ranged from the possibilities of having all data and feedback stored on one medical chip card to a lab inside a bus that allows end users to do all biometric tests close to home (eg, remark d7).

In the “usefulness” domain, a clear difference was seen in the type of issues identified by both groups concerning the relevance of the HRA. On one hand, designers were positive and thought that providing the HRA to this population was a good way of addressing health prevention options (eg, remark d9). On the other hand, some end users doubted the relevance for various personal reasons, and some also doubted its usefulness for the population as a whole (eg, remark e8).

Issues about the HRA’s usefulness were mainly identified by end users; they formulated those with either with positive or skeptical remarks. For example, positive remarks made by end users showed that they were satisfied because the HRA had brought a serious health problem to light (eg, remark e10) or because they were happy to have their good health confirmed. Skeptical participants indicated they felt the HRA was of limited value because the feedback presented only what they could have come up with themselves (eg, remark e9).

## Discussion

### Key Contributions

In this study, we assessed the output of a professional review by designers in a focus group compared to that of an end-user survey of a Web-based HRA with tailored feedback. Designers in their focus group identified 10 out of the 137 issues (7%) addressed by end users in their survey and brought to light many new issues.

To our knowledge, no previous studies have investigated whether designers in focus groups bring up similar issues as end-user surveys after participating in the same intervention. Despite designers’ full participation in the HRA as end users, our results show tendencies similar to studies in the fields of usability evaluation and text evaluation that compare the output of expert versus end user evaluations and the different methodologies applied [8,11,13]. The 7% overlap in the issues addressed in the evaluations of our study is, however, even lower than the 10-50% overlap found in these studies [8,11]. In their review of document evaluation methods, de Jong and Schellens [8] concluded that in expert evaluations many new issues are reported and that for expert evaluators the feedback of real readers often contains many surprising insights. The results of our study are in line with this conclusion.

The use of qualitative methods in this study provided insight into experiences of end users and designers. By analyzing their remarks using constructs of widely used theories on user satisfaction and technology acceptance, we were not only able to find that issues addressed by designers were dissimilar to those expressed in the end-user survey, but also to analyze differences in the nature of remarks made by both groups and pinpoint precisely which aspects of the intervention these differences pertain to. The analysis of these differences showed that although in both evaluations all high-level domains from the field of user satisfaction and technology acceptance were addressed, several lower-level topics were addressed in only one of the evaluations. When lower-level topics were addressed in both evaluations, often the nature of the associated remarks was different.

The differences in issues addressed show that end-user surveys should not be substituted for professional review in a focus group, despite the fact that both groups took on roles as HRA participants. On one hand, end-user surveys in the evaluation of HRAs give insight into immediate, practical opportunities for improvement, its perceived usefulness, and into whether the HRA meets or could be optimized to meet their expectations and enhance its adoption. These findings are in contrast with those of Lai [31] and of Lathan et al [32]. Lai found that end users were focused on system access and navigation, while we did not. Lathan et al found that end users were interested in their ability to use the system efficiently and effectively [32]. These differences in study results might be explained by the different foci of research (eg, research on system usability versus research on system acceptance and satisfaction) and suggest that the kind of evaluation method chosen greatly influences the kinds of issues an evaluation yields. Smilowitz et al concluded that nonexpert evaluations in real-life settings may uncover unforeseen problems introduced when the program is used [33]. Similarly, we observed various problems that affected end users’ perception of the system that were not discussed by designers. For example, the fact that confidence in the system is of major importance for the decision to implement health behavior recommendations could be deduced from answers by end users but was not discussed by designers. This and similar examples highlight the value of performing evaluations with end users in real-life settings to tackle unforeseen problems and that those problems cannot be found with designer focus groups.

On the other hand, designer focus groups yield insight into various aspects of proper design, and issues about the HRA’s user friendliness, efficiency, the display and completeness of the provided information, and organizational factors potentially impacting its adoption. Their discussion mainly focused on how the HRA can be improved in a broad variety of aspects (ie, idea generation) such as possibilities for (re)design, marketing and cost reduction, and its dissemination, while they presumed the usefulness of the innovation in general (ie, it is a good cause, it should be presented as a present). The fact that the designers emphasized the efficiency of the HRA contrasts with findings from Lathan’s study, which suggested that end users are more interested in their ability to use the system efficiently and effectively [32]. Our results are similar to those of Lai, who reported that experts tend to focus on information design [31].

In summary, the key difference between end-user surveys and designer focus groups is as follows. End users report on immediate experiences with problems and not their causes. In contrast, designers in focus groups discuss how to solve problems that have been encountered and formulate additional requirements for improving the HRA’s design. This difference was illustrated by how remarks by end users and designers were formulated. For example, end users formulated their remarks more like “I thought the feedback was…I would prefer…”, while designers formulated the same issue as end users as “the feedback should be presented so that…”

The implication of our study is that HRA evaluation can be performed only with end-user surveys, or with a combination of both end-user surveys and designer focus groups, and the choice should depend on the focus of the evaluation. Among the most significant advantages of evaluating HRAs by means of end-user surveys are their potentially wide reach, their low cost, that they provide the opportunity to quickly determine the perspectives of end users, and that they can be used periodically for comparisons over time. However, they often have low response rates and associated risks of bias and do not allow participants to elaborate on the issues addressed. Adding professional reviews with focus groups to the evaluation is a choice that depends on the information needed for optimization of the innovation and the available resources. This method is easy to administer and is a relatively quick and low-cost method for obtaining information about potential attitudes and experiences of participants. The data analysis, however, is often time consuming and the quality of the data collection is in part dependent on the skills of the research team performing the focus group. Combining both end-user surveys and designer focus groups in HRA evaluations might enhance its adoption and design, as both evaluations resulted in a different focus within a theoretical framework that has previously been shown to be useful in understanding system impact and actual usage behavior [3,34]. Whether this actually enhances adoption must be proven in future studies.

### Limitations

Various points for improving the HRA were identified from the issues addressed by end users and designers. However, from our study we are unable to determine whether the issues addressed in the designer focus group are valid nor how important they are. To explore this, these issues could be presented to end users to assess whether these issues actually influence utility and acceptance of the HRA. The added value of using one or both of the evaluation methods could be explored by implementing the suggested improvements in the HRA and studying the rates of adoption. It may also be useful to investigate whether some form of cross-fertilization is possible or whether the performance of designer focus groups can be enhanced by training.

The conditions of the two evaluation methods that were compared differed in various aspects from each other. The methods differed in the composition of the evaluator group, evaluation technique, and in the instructions given to the two groups in the evaluation. Therefore we are unable to comment on the causes of the differences found in the yield of both evaluations. Additionally, varying aspects of the evaluation techniques applied might provide different results. However, the goal was to compare the yield of the two methods in the way they are commonly used in practice to inform the optimization of new health technology innovations post-implementation. Our goal was not to compare them in a traditional experiment that aims to control these types of variations through methodology.

Although the designers in the focus group were specialized in different areas of design engineering, the study sample was essentially monodisciplinary. A group with varying disciplinary backgrounds might have delivered more comprehensive answers and shown different results. However, a disadvantage of multidisciplinary groups is that they might not speak the same language. Further research into evaluating HRAs post-implementation with a more multidisciplinary group might, however, prove valuable.

Another shortcoming of our study is the fact that the response rate to the free-text field was low when compared to other satisfaction surveys [35,36]. The explanation is likely that filling in the free-text field requires additional time and effort. Respondents had already filled in the structured part of the evaluation questionnaire, and they may have felt that this already covered all of their remarks on the HRA. Also, end users in the survey could have interpreted the question preceding the open-text field in two different ways, that is, as an invitation to comment on the HRA in general or on the survey itself. However, qualitative analysis of the end user remarks showed that most end users interpreted the question as an invitation to comment on the HRA in general; only 2.9% of the remarks were about the survey itself. Nevertheless, we cannot ignore that specific groups of end users left the free-text field blank.

Finally, categorization of the designers’ remarks was performed after all end user responses had already been categorized, rather than doing this simultaneously. However, this should not have influenced the interpretation of their remarks, as we kept going back to the data of both end users and designers in our iterative process if changes were made in our categorization or framework. Subsequently, 1 researcher rather than 2 researchers grouped together identical issues and systematically compared the issues identified by end users and designers to limit the workload. However, 2 researchers independently assessed these final issues and made changes where necessary. These 2 researchers validated the comparison of issues revealed by the end-user survey and designer focus group.

### Conclusions

Most post-implementation evaluations of health promotion programs, including eHealth applications, are performed with end users. In this study, professional review by a focus group of industrial designers of a Web-based HRA proved that this type of evaluation mainly yields new issues when compared to the feedback provided by end users in a Web-based survey. The end-user survey gave insight into the extent to which expectations and needs of users were met and suggested how the HRA could be improved to enhance its adoption in practice. Designers in the focus group gave more constructive criticism and provided recommendations to improve the HRA design and its marketing. They focused not only on potential problems but also made suggestions for (re)design, marketing, costs, and opportunities for future HRAs. We recommend that end-user surveys not be substituted for professional review in a focus group. Instead a combination of both methods in the evaluation of HRAs and other health promotion programs may prove more advantageous.

## Multimedia Appendix 1

Characteristics and interpretations of the codebook used to structure the data.

## Multimedia Appendix 2

All equivalent issues identified by end users and designers about the Web-based HRA with tailored feedback.

## Multimedia Appendix 3

Illustrative remarks within domains to illustrate the differences in yield of the two evaluation methods.

## Abbreviations

CDSS: computerized decision support system
DUT: Delft University of Technology
HRA: health risk assessment
