# Supplementary material for: Evaluating a Web-Based Health Risk Assessment With Tailored Feedback: What Does an Expert Focus Group Yield Compared to a Web-Based End-User Survey?
Source: J Med Internet Res. 2014 Jan 2;16(1):e1. doi: 10.2196/jmir.2517 (PMC3906664; doi:10.2196/jmir.2517)
Supplement: Supplementary file 1 [file jmir_v16i1e1_app1.pdf]

Multimedia Appendix 1. Characteristics and interpretations of the codebook used to structure the data

The codebook is an adapted version of the domains and topics that are described in Table 1 of Wixom and Todd 2005 (Table 1, p 88). In this table all domains and topics of user satisfaction and technology acceptance theories are included. The table below describes various domains (Wixom and Todd use the term "external variables") and topics (Wixom and Todd use the term "instrument characteristics"). If a comment did not pertain a topic in the original codebook, topics were copied from other domains or an appropriate new topic was added to the codebook during a consensus meeting. Definitions are adapted from the framework created by Wixom and Todd, 2005; and Bailey and Pearson, 1983. Definitions were specified further to the HRA in the current study and in the study of Vosbergen and Laan et al, 2012 during qualitative analysis.

Table 3. Characteristics and interpretations of the codebook that was used to categorize all end user and designer remarks alike.

Domain	Topic	Explanation
**Ease of use**		***Degree to which users believe that using the HRA is effortless.***
	Easy to use	*The HRA effectively fills users' needs and is fast and free of errors.*
	User-friendly	*The HRA is pleasant to use and easy to learn.*
**Information Quality**		***Users' perceived quality of the information given by the HRA.***
	Accuracy	*Users' perception that the information is correct.*
	Completeness	*The degree to which the HRA provides all information perceived as necessary by the user.*
	Format	*The layout and display of the information throughout the entire web portal.*
	Language[b]	*Users' ability to understand the language used in the HRA.*
	Precision	*The variability of the output information from that which it purports to measure.*
	Volume	*The amount of information conveyed to users.*
**Organizational factors**		***Influence of the organization, procedures, and choices on the quality of the HRA.***
	Communication[a]	*The availability of correct information before using the HRA.*
	Organizational competition	*Congruence between the assessments and feedback provided by the system and an external health professional.*
	Error recovery	*The way the staff and organization manages errors as consequence of the service delivered by the company behind the HRA.*
	Data security	*The safeguarding of data from misappropriation or unauthorized access, alteration, or loss.*
	Documentation	*The recorded description of an information system. This includes formal instructions to the user and to program staff about the HRA.*
	Management	*Experiences with or opinions about the choices made by any management level above the users' own level that influences how the HRA is perceived, like the way the HRA is implemented and what the costs for the HRA are.*

	Time[a]	*The availability of the evaluation questionnaire at a time suitable for its use.*
**Outcome expectations**		***Congruence between users' expectations and actual situation with regard to using the HRA and the feedback provided by the system.***
	Accuracy[a]	*Users' perception that the provided feedback is congruent with their expectations about their health.*
	Confidence in the system	*Users' feelings about the reliability of the HRA and the feedback provided by the system.*
	Feelings of control	*Users' perceived power to regulate/influence the feedback provided by the system.*
	Expectations	*Users' expectations of the HRA.*
	Health effects	*Users' (expected) changes in lifestyle or other health-related issues as a result of using the HRA.*
**Service quality**		***Users' perceived quality of the service delivered by professionals associated with the HRA.***
	Attitude of program staff	*The way users perceive the attitude of the staff towards users and their health experiences.*
	Communication with the staff that is associated with the HRA	*The way information is exchanged among users and staff.*
	Means of input of the HRA	*The method and medium by which users receive services from staff and/or the system and the perceived usefulness of this service.*
	Processing of change request	*The manner, method, and required time the staff respond to users' requests.*
	Relationship with program staff	*The method and manner of interaction between users and staff.*
	Schedule of products or services	*The timetable for system output, services, and procedures.*
	Staff support[b]	*The service and help offered by the staff associated with the HRA.*
	Technical competence of program staff	*The skills and expertise of the staff.*
	Response time	*The time between users' requests for service or action and response to these requests.*
**System quality**		***Users' perceived quality of the web-based component of the HRA.***
	Accessibility	*The degree to which the system is accessible to its users.*
	Efficiency	*The rate or speed at which the system enables users to accurately and successfully complete a task.*
	Errors[a]	*The methods and policies governing correction and rerun of incorrect system output*
	Flexibility	*The capacity of the system to change or adapt in response to new conditions, demands, or circumstances.*
	Language	*The set of vocabulary, syntax, and grammatical rules used to interact with the system.*
	Tailoring[b]	*The ability of the system to take user characteristics into account.*
	Timeliness	*The availability of system's output at a time suitable for its use.*
**Usefulness**		***General usefulness of the HRA for its users.***
	Relevancy	*The degree of congruence between users' needs and requirements and what the HRA provides.*
	Usefulness	*The extent to which the HRA actually helps to solve users' problems.*

[a] Topic is copied from another domain

[b] Concept is added to the codebook

Note: Topics that were part of the original codebook but not used during analysis are not described in the above table. These characteristics were integration (system quality), vendor support, time required for new development (service quality), understanding of systems, feelings of participation, degree of training (outcome expectations), priorities determination, charge-back method (organizational factors), reliability and currency (information quality).
