# Supplementary material for: Evaluating a Web-Based Health Risk Assessment With Tailored Feedback: What Does an Expert Focus Group Yield Compared to a Web-Based End-User Survey?
Source: J Med Internet Res. 2014 Jan 2;16(1):e1. doi: 10.2196/jmir.2517 (PMC3906664; doi:10.2196/jmir.2517)
Supplement: Supplementary file 2 [file jmir_v16i1e1_app2.pdf]

Multimedia Appendix 2. All equivalent issues identified by end users and designers about the web-based HRA with tailored feedback.

Table 4. Issues that were identified by both end users and designers.

**Issue number**	**Domain**	**Topic**	**Illustrative remark – end user**	**Illustrative remark - designer**
**1**	**Ease of use**	*Easy to use*	*It would have been practical if the outcomes could be printed in a normal manner.*	*What's most important to me is that you can print the advices and re-read the interpretation of the values.*
**2**	**Ease of use**	*Easy to use*	*When filling in the questionnaire it would have been practical when the back button could be used.*	*Did you think it was unpleasant that you did not just get the complete questionnaire but that you had to back to change your question?*
**3**	**Information quality**	*Accuracy*	*Answering the questions in the HRA takes a lot more time than was indicated. In my opinion, this should have been clear so that you can bear this in mind.*	*Yes, that you know how long you have to reserve for this process.*
**4**	**Information quality**	*Completeness*	*When the results are shown, there's not enough mention of reference materials… the results is 4, but on which scale?... what's good and what isn't, and when or at what score should I be concerned?*	*What is required is that you get a printout of all the recommendations along with the referrals to a website or an individual. Along the Lines of, what is good, what isn't, and what does it actually mean?*
**5**	**Information quality**	*Format*	*I thought the feedback was very inconveniently arranged. Needing to click all the time leads the attention from important matters. I would personally have found it more pleasant when the most important conclusions of the things that can be better/different would be summarized and that more information could be found at the concerning topic.*	*Or, at least, presented in a better manner and also short, concise and clear and if you want more information, that that is also possible.*
**6**	**Information quality**	*Format*	*Because all communication is written (in other words, in the system), it's easy to disregard any potential recommendations.*	*No, but the results also didn't really provide the motivation to go into them at length.*
7	**Organizational factors**	*Data security*	*Please sent me a confirmation that my data is deleted from the internet (with respect to privacy of the data).*	*I have a marginal comment about that: I understand when individuals are wary about their health data with their employer… I know, it is anonymus.*
**8**	**Outcome expectations**	*Confidence in the system*	*Results are based on the answers that are filled in, and not on blood and urine tests.*	*But most of the advices were based on that evaluation form and not at the blood tests.*
**9**	**Service quality**	*Means of input of the HRA*	*I thought it was not practical to have to travel such a distance for the blood test. I would have liked if it had been at the office, because I was outside in 10 minutes.*	*Yes, there were a lot of addresses where I could go, but all of them were outside (home city), in Amsterdam.*

**10**	**Service quality**	*Relationship*	*The staff members are competent and friendly.*	*I thought that was pleasant [the visit].*
