# Supplementary material for: Evaluating a Web-Based Health Risk Assessment With Tailored Feedback: What Does an Expert Focus Group Yield Compared to a Web-Based End-User Survey?
Source: J Med Internet Res. 2014 Jan 2;16(1):e1. doi: 10.2196/jmir.2517 (PMC3906664; doi:10.2196/jmir.2517)
Supplement: Supplementary file 3 [file jmir_v16i1e1_app3.pdf]

Multimedia Appendix 3. Illustrative remarks within domains to illustrate the differences in yield of the two evaluation methods.

Table 5. Examples of end-users' remarks and designers' remarks to illustrate the differences within domains of the coding scheme.

**Domain**	**End-user remark**	**Designer remark**
Ease of Use	It is troublesome that you have to scroll constantly in the feedback report and to continuously have to click the explanation-icons. (Easy)	If you look at the Dutch voters guide website Kieswijzer, then you can change your answer by going to the menu of that question. (Easy)
	The questionnaire is really extensive, and in particular the questions regarding the incidence of family diseases are not always easy to answer." (Easy)	And you also don't know, if I have a choice: Alcohol. If I click no, then I probably go to the next question. If I click yes, do I get another 100 questions? […]. You just don't know, so I will click 'no'. (Friendliness)
		And then the feedback report. I thought that there was a lot of information and you could click on and on and then somewhere there appears information again. Then I rather have it on paper, it is a lot of course. (Friendliness)
Information quality	After receiving the results, I didn't really understand the feedback. I got the advice to eat healthier. I actually already started eating healthier food some time ago (and I have indicated this in the questionnaire). Still I received this advice, but I wouldn't know what else to do. (Completeness)	Just explain that there is one way to complete things. People should just… you just have to guide them, because that's the most useful. It's also the most efficient. (Completeness)
	My general practitioner was really unsatisfied with how the HRA works, there is no explanation given about what has been tested precisely etcetera. Due to these results, there have been, according to my general practitioner, needless blood test via STAR. (Accuracy)	What you can also do, you have those five or six subjects in the feedback report, you can also have a compass per health subject. Because then you'll see a compass at the left top, this compass does this (mimics a compass pointer): for this subject you go wrong and for this one you go well. (Format)
	A feedback report with more specificity would have been more clear…. What one can or cannot do and must do. (Format)	Make it a little bit more personal, instead of relating it to some kind of standard. Because now you get: this is healthy, this is you, you fail in this, or you fail on that. But just say something like: This is personal, this is what you are now, and this is what you could do, this, that, etcetera. Instead of, relating it to the mass, you are wrong here and there and there. (Format)
	The feedback report of the HRA got across fiercer on me than it was in fact. It is a good realization and certainly a good provocation to take action. Nevertheless, I had preferred a few things to be expressed more subtle. (Format)	
Organizational factors	My general practitioner disagrees with the results concerning a high cholesterol due to a difference in the interpretation of the numbers, that's why in your case I am in the risk group and according to the general practitioner not. Furthermore, measuring the blood pressure with an electrical measurement device gives other results than measurement by hand. That is why,	If I were to do it, I would just like my house style to be consistent. (Communication)

	according to the HRA, I belong to the risk group while according to my general practitioner, I do not (Competition)	
	Especially with sending the tools it went wrong several times. I didn’t have tools for the urine examination etcetra when I should have been at the examination already. Redirecting it didn’t work immediately also. (Error)	You have to get someone excited, so you can say that it is a gift to the people. […] And that you, for example, mention somewhere; this package costs X euro, but the government and the employer believe it is important that…. Just a story that they know that it is no garbage, but that it is actually really valuable and that they get it for free. (Communication)
		It is likely that it can be cheaper, so than you will still come.. If you just demand: X euro, than you will obtain that target right? (Management)
Outcome expectations	The feedback report is clear, but I am wondering whether one can conclude from these limited tests how healthy I live and what my physical condition is. (Confidence)	Because I knew they would give this kind of advices, if I would have filled it in like this [..]. So one expects more. (Expecatations)
	I will go to the general practitioner because I am ‘seriously off-track’. Let’s see what he says. My family members were yet rather frightened from the word ‘seriously’. (Confidence)	It gave me the feeling that… I thought: Yes I will eat more healthy, but when I will start doing so? And than there was such an…again.. (laughs). That gave me a negative feeling to indicate that. (Health effects)
	I realize you want the phrasing of the questions to be as clear as possible. In a number of cases, the answers are oversimplified. The actual situation is sometimes far removed from the possible answers and consequently the results also give a different (more negative) picture. (Feeling of control)	
	The examination does not have any relation to my work activities. Work related problems/complaints are insufficiently covered because of this. (Expectations)	
Service quality	Viewed apart it is great that blood, cholesterol, bloodpressure and the like are examined, but it is still a random indication (Means of input for the HRA)	That’s when I thought, I will first fill in the questionnaire before I set to work with measuring my blood pressure. Or was it that I first waited for the lab box, I don’t know anymore. I first want to know everything, you know, to get an overview of what I have to do. (Means of input for the HRA)
	During a face-to-face talk you could have given a lot more information and clarified things, and also have had a more thorough physical examination. (Means of input for the HRA)	I was also afraid that I would be called. [..] If there is something really wrong, we will call you. (Means of input for the HRA)
	I thought the biometric evaluation visit was quite basic. All of the procedures were carried out in a rather impersonal way and at breakneck sped. It felt a little bit like a production line. (Relationship)	With the card you can activate your account and you also have to use the card to perform your measurements (has the card in his hands). Everything you do is stored on your card and if you log in with the card, your data will automatically be stored on the website, on your account. And the parameters, the outcomes of the blood tests are eventually also stored on your card and with this card you can go to the general practitioner and he explains to you what to do. (Means of input for the HRA)
System quality	After I received my results I wanted to	No, in some cases the questions aren’t smart.

	examine my answers, only this wasn't possible anymore (it would be convenient if this was possible). (Accessibility)	Because when I filled in 'no' at the psychological questions, then as a last question I got: have you answered one of the previous questions with 'yes'? Well, you can refer that, right. (Efficient)
	The reasons why my results are orange are vague to me. I indicated that I broke a vertebra in an accident. What has this got to do with osteoporosis? I am deaf in my left ear since my birth. Why do I have to come over to talk about my hearing? I also had to come over because my sight is suboptimal. But that is the reason why I wear glasses. (Flexibility)	You can immediately register at the internet. You immediately receive a login, so you can immediately fill in the questionnaire and only if you have completed the questionnaire you receive the home measurement tools. (Efficient)
	There isn't a possibility to get into the personal situation in detail. (Tailored)	
	I missed men and women directed questions in the questionnaire. (Tailored)	
Usefulness	I don't think everybody needs to have an HRA. It upsets people more than anything else, and doesn't give any guarantees at all. It's useful for (hereditary) diseases in the family. The question remains as to whether this should be done through the employer. (Relevance)	It is ideal to just, as an employee, so to speak not with your Christmas box, but… So you get it from your employer, you think: 'Hey that employer has exerted himself, it is a subsidized thing, I really think it is a good cause. (Relevance)
	I don't know whether I would participate a next time (when is the next time??). I prefer to consult my general practitioner and going to the physician-lab to draw blood. (Relevance)	Feedback; that is my wish. That the feedback is the goal. Because that is what you have act on, isn't it? (Usefulness)
	Loss of time, drawing conclusions based on length, weight and a few simple internet-based questions. (Usefulness)	
	A polyp has been removed from my intestines on two different occasions. According to the specialist, one of these would certainly have become malignant. (Male, Age: 46, Usefulness)	
